# 86057 High Screening Efficacy Using Wearable Seismocardiography to Identify Aortic Valve Disease Patients, Potential to Tailor MRI Exams to Patient Needs

**DOI:** 10.1017/cts.2021.640

**Published:** 2021-03-31

**Authors:** Ethan M.I. Johnson, Alex J. Barker, Michael Markl

**Affiliations:** 1 Northwestern University; 2 University of Colorado, Anschutz Medical Campus; 3 Northwestern University

## Abstract

ABSTRACT IMPACT: A single seismocardiography (SCG) parameter has been shown to accurately classify aortic valve disease (AVD) status in healthy controls and AVD patients. This could support development of SCG as a quick, inexpensive screening tool to better tailor MRI examination to patients’ needs. OBJECTIVES/GOALS: MRI is used commonly for monitoring of aortic valve disease (AVD), but it has high costs. We hypothesize that energy in seismocardiograms (SCG)’‘ signals from chest surface vibrations’‘ is different between healthy controls and AVD patients, and we evaluate potential efficacy of using SCG to recommend MRI only for patients with flow abnormalities. METHODS/STUDY POPULATION: With IRB approval, 45 healthy control subjects (47 ±18years, 18 female) and 9 patients (63 ±16years, 2 female) with aortic valve disease history and known flow abnormalities were recruited. SCG signals were acquired supine, immediately prior to MRI of thoracic aortic blood flow at 1.5T with a time-resolved phase contrast (4D Flow) sequence.

The SCG was processed to calculate late-systole high-frequency (120-240Hz) RMS energy. MR velocity images were analyzed to measure peak velocity and trace pathlines of flow.

Screening efficacy of the SCG energy metric was assessed, with hypothesis testing for differences in energy level distributions between controls and patients, and receiver-operator characteristic (ROC) analysis was used to calculate rates of correct/incorrect classification of disease. RESULTS/ANTICIPATED RESULTS: Healthy subjects had coherent flow pathlines through the aortic arch and mid-ascending aorta peak velocities of 106 ±21cm/s (cohort mean ±standard deviation). All valve disease subjects had flow abnormalities, such as jetting flow near the valve or swirling through the arch, as visualized by pathlines. Patients’ peak mid-ascending aorta velocities were 167 ±69cm/s. The SCG energy for healthy controls was significantly different than that of valve patients (-5.6 ±0.3dBmm/s/s vs. -4.0 ±1.2dBmm/s/s respectively; p<0.001). Thresholding SCG energy to distinguish patients from controls correctly classifies subjects with a high true-positive rate and low false-positive rate. The ROC for this classification has area-under-curve 0.956. DISCUSSION/SIGNIFICANCE OF FINDINGS: A high potential screening efficacy was observed using a single, linear SCG metric to identify AVD patients with flow abnormalities. If used to complement MRI surveillance protocols for AVD, this method has potential to serve as a quick, inexpensive tool for better tailoring MRI exams to patient needs.

